# How to frustrate a plant pathogen

**DOI:** 10.1111/tpj.70971

**Published:** 2026-06-06

**Authors:** Gregory Knight, Jonathan Heddle, Adam R. Bentham

**Affiliations:** ^1^ Centre for Programmable Biological Matter, Department of Biosciences Durham University Durham DH1 3LE UK

**Keywords:** plant immunity, effectors, bioengineering, structural biology, frustration, biophysics, protein design

## Abstract

Sequence‐unrelated but structurally similar (SUSS) effector families represent a distinctive evolutionary strategy among plant pathogen virulence proteins. Within families such as MAX, LARS and RALPH effectors, individual proteins maintain nearly identical three‐dimensional folds despite minimal sequence identities, whilst targeting functionally diverse host cellular processes. This decoupling of structural conservation from functional specificity challenges traditional precepts of the classic structure–function paradigm and reveals how pathogen effectors exploit stable protein scaffolds as platforms for rapid functional diversification through extreme sequence variation. Comparative structural analyses suggest that surface frustration, regions of local energetic instability essential for fold flexibility, may be conserved across SUSS family members despite sequence divergence. This conservation creates potential vulnerabilities that could be exploited for resistance engineering. Rather than targeting individual effector‐host interactions, frustration‐guided design of molecular sponges, synthetic integrated domains or proteome degradation warheads could potentially neutralise entire SUSS effector families. This review explores the mechanisms of functionalisation by SUSS effectors and suggests strategies combining structural genomics, surface frustration analysis and AI‐driven protein design for developing broad‐spectrum resistance against major classes of plant pathogen effectors.

## Significance Statement

Plant pathogens deploy large families of structurally related but sequence‐diverse effector proteins to manipulate host cells, allowing them to evade immune recognition whilst maintaining the capacity to target diverse host processes. This review discusses surface frustration as a conserved biophysical property of these effector families that may be exploited as a shared vulnerability, proposing that AI‐driven protein design targeting these structural features could enable broad‐spectrum resistance effective against entire effector families rather than single proteins.

## INTRODUCTION: SUSS EFFECTORS DO NOT FOLLOW A CLASSICAL STRUCTURE–FUNCTION PARADIGM

Secreted virulence proteins (effectors) are essential for infection employed by plant pathogens to facilitate the colonisation of a host (Bentham et al., 2020; Dodds & Rathjen, 2010; Jones & Dangl, 2006). A pathogen can secrete a cocktail of hundreds of effectors into a host during infection, targeting immune signalling pathways, cell metabolism or homeostasis, with their structure largely dictating their function (Khan et al., 2018; Wilson & McDowell, 2022). The relationship between structure and function has long been a central tenet of phytopathogen effectors, with a general assumption that protein fold predicts molecular activity (Orengo & Thornton, 2005). However, some effector families systematically violate the fold‐to‐function precept of the structure–function paradigm. These sequence‐unrelated but structurally similar (SUSS) effector families conserve three‐dimensional folds whilst targeting functionally diverse host proteins, raising intriguing questions about the mechanisms enabling such structural constraint coupled with functional plasticity (de Guillen et al., 2015; Franceschetti et al., 2017; Mukhopadhyay et al., 2025; Seong & Krasileva, 2023). In this review, we examine characterised SUSS effector families across filamentous pathogens, explore proposed mechanisms underlying their evolutionary success and consider how biophysical analyses of conserved structural features, particularly surface frustration patterns (Derbyshire & Raffaele, 2023), might reveal vulnerabilities exploitable for engineering broad‐spectrum resistance against entire effector families.

## STRUCTURAL CONSERVATION MAY FACILITATE FUNCTIONAL DIVERSITY IN PATHOGEN EFFECTORS

The discovery of SUSS effector families has revealed a distinctive evolutionary strategy. Unlike most protein families where structural similarity reflects shared ancestry and function (Orengo & Thornton, 2005), SUSS effectors demonstrate how conserved three‐dimensional folds can serve as stable platforms for extensive functional diversification (Seong & Krasileva, 2023). The paradigm of SUSS effectors was first noted through early structural studies (de Guillen et al., 2015); however, advances in protein structure prediction have since enabled the exploration of this phenomenon at the wider genomic scale (Mukhopadhyay et al., 2025; Seong & Krasileva, 2021, 2023).

SUSS effectors often exhibit three characteristics: first, family members share nearly identical protein folds with backbone TM‐scores typically above 0.5 (Seong & Krasileva, 2023); second, these protein families show no detectable sequence similarity, often with pairwise identities below 20% (de Guillen et al., 2015); and third, despite structural conservation, individual family members target functionally diverse host proteins and cellular processes (Franceschetti et al., 2017; Wilson & McDowell, 2022; Wirthmueller et al., 2013). These characteristics place SUSS effectors at the extreme edge of the classical structure–function landscape, revealing a decoupling between structural similarity and functional inference.

The phenomenon appears widespread among filamentous plant pathogens. Computational structural genomics surveys have identified extensive SUSS families across fungi and oomycetes, with the largest including hundreds of members within single pathogen species (Mukhopadhyay et al., 2025; Seong & Krasileva, 2023). This pattern can be exemplified based on experimental structure determination of three major families: MAX effectors from *Magnaporthe oryzae* share β‐sandwich architectures but engage different resistance proteins and virulence pathways (de Guillen et al., 2015); LARS effectors from *Leptosphaeria maculans* maintain disulphide‐stabilised folds whilst interacting with distinct *Brassica* immune receptors (Blondeau et al., 2015; de Guillen et al., 2015); and RALPH effectors across *Blumeria graminis* preserve RNase‐like structures despite many abandoning catalytic activity for diverse non‐enzymatic functions (Cao et al., 2023; Pennington et al., 2019; Seong & Krasileva, 2023).

The MAX family members, such as the well‐characterised AVR‐Pik and AVR‐Pia effectors, share a compact six‐stranded β‐sandwich architecture, with structural analyses showing Cα root‐mean‐square deviations below 1.5 Å despite sequence identities seldom exceeding 20%. Studies of host target interaction have primarily demonstrated interaction with heavy metal associated (HMA) domains in host proteins (Bentham et al., 2021; Maidment et al., 2021; Oikawa et al., 2024; Sugihara et al., 2023). These studies demonstrated that AVR‐Pik and AVR‐Pik‐like (APikL) effectors have different HMA specificity due to their sequence diversity, and this has been correlated with host target range of *Magnaporthe* strains (Bentham et al., 2021; Maidment et al., 2021). AVR‐Pia similarly targets HMA domain‐containing rice H(I)PP proteins, with structural characterisation of the AVR‐Pia/OsHPP09‐HMA complex revealing the molecular basis of binding (Maidment et al., 2025). Strikingly, the MAX effector Avr‐Piz‐t abandons HMA targeting entirely, instead suppressing host immunity through interaction with the RING E3 ubiquitin ligase APIP6 (Park et al., 2012). That structurally near‐identical proteins engage such functionally distinct host proteins illustrates the SUSS principle within a single well‐characterised effector family.

LARS effectors from *L. maculans* demonstrate SUSS characteristics in smaller protein scaffolds. Effectors such as AvrLm4‐7 and AvrLm5‐9 adopt nearly identical disulphide‐stabilised αββ folds with Cα RMSDs around 1.2 Å, despite sequence identities below 15% (Blondeau et al., 2015). These effectors target distinct aspects of host cellular function, with AvrLm4‐7 additionally capable of suppressing immune responses triggered by unrelated effector families through unknown host protein interactions (Lazar et al., 2022).

Fungal RALPH effectors as found in powdery mildew (*B. graminis*), characterised by their RNase‐like folds despite lacking catalytic activity (Cao et al., 2023), extend SUSS principles to enzymatic scaffold repurposing (Seong & Krasileva, 2023). These proteins adopt canonical αβ architectures with TM‐scores above 0.6 compared to authentic fungal RNases (Seong & Krasileva, 2023), yet perform entirely non‐enzymatic functions through targeting diverse host cellular machinery (Harris et al., 2023; Pennington et al., 2019; Xu et al., 2019). Some localise to chloroplasts to interfere with photosynthetic processes (Xu et al., 2019), whilst others act in the nucleus to manipulate transcriptional programs or metabolic pathways (Harris et al., 2023).

The functional diversity of host protein targets across SUSS effector families contrasts with their limited recognition by individual plant immune receptors (Na & Gijzen, 2016). Whilst immune surveillance proteins typically recognise only one or few members of each SUSS effector family, this selectivity likely reflects the challenge of tracking rapidly diversifying effectors rather than evolutionary pressure on effectors to prevent recognition through interactions with specific immune receptors (Derbyshire, 2020; He et al., 2020; Khan et al., 2018). This pattern suggests that SUSS effector diversification successfully evades family‐wide immune recognition whilst maintaining the ability to manipulate diverse host cellular processes.

The SUSS effector evolutionary pattern may reflect selection under a fundamental constraint facing pathogens: how to maintain and generate new virulence functions to counter host defences whilst ensuring structural integrity required for protein folding, secretion and delivery. The RALPH effectors are an exemplar of this strategy, utilising an evolutionarily constrained RNase scaffold that has often lost enzymatic activity but allows for functional innovation through surface variation (Derbyshire & Raffaele, 2023). However, this balance between structure and surface variability may reveal exploitable vulnerabilities for resistance engineering.

## SUSS EFFECTOR CHARACTERISTICS CAN BE EXHIBITED AND FUNCTIONALISED AT THE DOMAIN LEVEL

**Figure 1:**
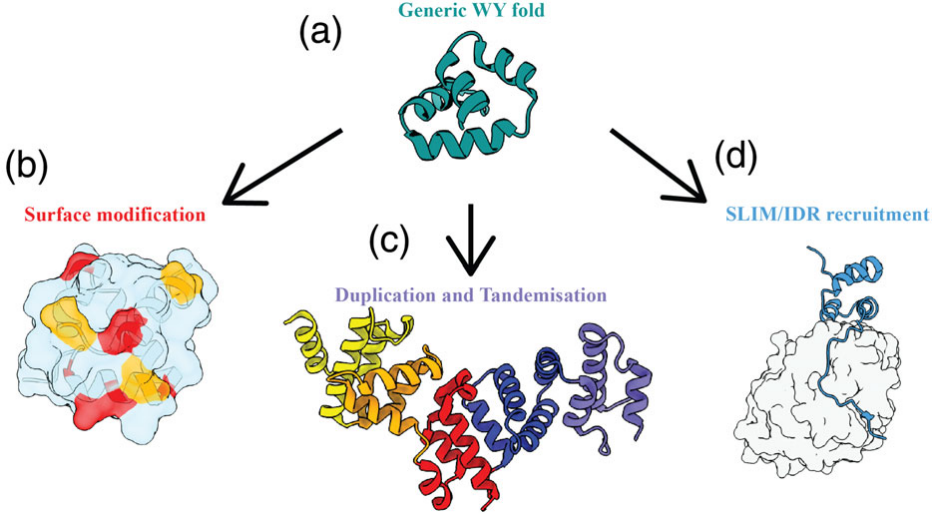
Diversification allows SUSS families to achieve a range of functionalities whilst maintaining the core fold architecture. (a) SUSS effectors achieve diversity through three main methods, exemplified using a generic WY fold exemplar: Surface modification, duplication and tandemisation and SLiM/IDR recruitment. (b) Surface modification can introduce diverse local biochemical environments. Local amino acid changes can provide both beneficial changes for effector functionality (Red) as well as changes to evade detection by host immune recognition (Yellow). (c) Asymmetrical tandemisation creates large, novel structures from a simple single fold. Different colours represent different core fold subunits, with tandemisation often achieved through asymmetrical repeats. (d) SLiM/IDR recruitment offers plug and play potential for effectors introducing short motifs crucial for binding. The effector is shown in blue whilst the target is shown in grey. The core motif of the effector can directly bind; however, the acquired SLiM enables increased specificity to the host target.

SUSS effectors were first characterised in phytopathogenic fungi (de Guillen et al., 2015), but the concept appears to be ubiquitous throughout filamentous pathogens (Seong & Krasileva, 2023). RXLR‐WY effectors from *Phytophthora* species (herein referred to as WY effectors), first structurally characterised by Boutemy et al. (2011), have recently been recognised as exemplifying the SUSS phenomenon (Mukhopadhyay et al., 2025). These WY effectors adopt a conserved four‐helix bundle architecture stabilised by buried hydrophobic cores centred on tryptophan (W) and tyrosine (Y) residues (Franceschetti et al., 2017; Wilson & McDowell, 2022; Wirthmueller et al., 2013) with a subclass of this family also incorporating a conserved leucine (L; LWY effectors) (Ye & Ma, 2016). Despite backbone root‐mean‐square deviations below 1 Å between the WY domain core of family members demonstrating high structural similarity, sequence identities frequently fall below 20%, creating the sequence‐structure disconnect that defines SUSS families (Seong & Krasileva, 2023). Interestingly, duplication of WY domains within the effector architecture is often observed (Franceschetti et al., 2017), allowing the pathogen to use WY domains as building blocks to create new functions and greatly expand on the surface that can be functionalised whilst still maintaining a regularly identifiable structural core (He et al., 2019; Li, 2025) (Figure 1a). Previous studies have observed that this ‘beads‐on‐a‐string’ approach represents an elegant evolutionary pathway where pathogens can ‘mix and match’ locally conserved folds to build globally diverse and complex effectors, rapidly expanding their functional toolkit (Franceschetti et al., 2017; He et al., 2019; Li, 2025).

Within this conserved structural framework, WY effectors from *Phytophthora* species have diversified to target functionally distinct host cellular processes. AVR3a11 interferes with host E3 ubiquitin ligases to disrupt protein degradation pathways and suppress immune responses (Bos et al., 2010). PexRD2 orchestrates host cell physiology through interfering with MAPKKK immunity related signalling (King et al., 2014). Pi04314 exploits the same WY scaffold to target protein phosphatase 1c (PP1c), using a C‐terminal KVxF motif to mimic endogenous regulatory proteins and redirect nuclear phosphatase activity (Bentham et al., 2024; Boevink et al., 2016). PSR2 from *Phytophthora sojae* demonstrates architectural elaboration through seven concatenated WY/LWY repeats that collectively target RNA silencing machinery by binding double‐stranded RNA‐binding proteins (He et al., 2019).

It is clear WY effectors can have diverse global folds despite the highly homologous individual folds at the domain level. This may represent an alternative example of the SUSS effector strategy, where local structural conservation combines with high sequence variation to create global structural diversity that permits new functions.

## MOLECULAR MECHANISMS UNDERLYING SUSS EFFECTOR DIVERSIFICATION

The conservation of three‐dimensional architecture within SUSS effector families, despite extreme sequence diversification and functionality towards different host protein targets, reveals how structural constraint and evolutionary flexibility can be reconciled through distinct but complementary molecular mechanisms that permit exploration of sequence and function space within a conserved fold space.

Surface loop variation represents the primary mechanism for functional diversification within SUSS families (Derbyshire & Raffaele, 2023). Comparative structural analysis shows that sequence changes concentrate in peripheral loops and terminal regions whilst hydrophobic cores remain highly conserved (Figure 1a). In MAX effectors, variable surface patches determine binding specificity to different host protein families despite identical β‐sandwich scaffolds (de Guillen et al., 2015; Lahfa et al., 2024). Similarly, LARS effectors achieve distinct host protein recognition through surface‐exposed residue changes whilst preserving their disulphide‐stabilised αββ architecture (Blondeau et al., 2015; Lazar et al., 2022). This spatial segregation of conservation and variation in SUSS effectors suggests that surface regions, despite sequence‐level divergence, may retain conserved biophysical properties such as charge distribution, flexibility or shape complementarity, that may enable compatible interfaces with structurally diverse host proteins (Derbyshire & Raffaele, 2023; Shahmoradi et al., 2014).

Domain duplication and concatenation expand the functional capacity of scaffolds that have demonstrated virulent functions for engaging multiple host protein targets simultaneously (Aluru & Singh, 2021; Li, 2025) (Figure 1b). WY proteins like PSR2 contain seven tandem repeats, each maintaining the canonical four‐helix bundle whilst collectively creating an extended architecture capable of engaging multiple components of RNA silencing machinery (He et al., 2019). Variants that destabilise the structural core are likely filtered by selection, resulting in the observed retention of fold stability alongside massive sequence diversity and expanded interaction space through WY domain repetition, enabling the targeting of a wide variety of host proteins.

Short linear motif (SLiM) acquisition provides a rapid mechanism for gaining new host protein interaction capabilities (Chepsergon et al., 2022; Elkhaligy et al., 2022; Salasini et al., 2025) (Figure 1c). These minimal sequence elements, typically 3–10 amino acids, can be inserted into flexible regions without disrupting fold integrity whilst enabling binding to entirely different host protein families. Pi04314's KVxF motif enables protein phosphatase 1c binding, transforming a basic WY scaffold into a phosphatase regulator (Bentham et al., 2024; Boevink et al., 2016). PexRD54 carries an autophagy‐interacting motif (AIM) that links the effector to host autophagy machinery rather than other cellular processes (Dagdas et al., 2016). The modular nature of SLiMs allows functional variation and host target adaptation (Elkhaligy et al., 2022), whilst allowing a greater degree of sequence diversification of the core fold without affecting host target interaction.

Intrinsically disordered regions (IDRs) flanking structured domains in SUSS effectors contribute additional flexibility for host protein recognition and subcellular targeting (Chepsergon & Moleleki, 2023) (Figure 1c). These regions provide conformational plasticity that may enable context‐dependent binding modes or serve as platforms for evolving new SLiMs directed towards different host protein families, a model which has been shown in viral systems (Glavina et al., 2022; Holehouse & Kragelund, 2024). RALPH effectors demonstrate this principle, where disordered termini facilitate subcellular targeting to different cellular compartments (chloroplasts vs. nucleus) whilst preserving the RNase‐like structural core (Cao et al., 2023; Franceschetti et al., 2017). The dynamic nature of IDRs may also give rise to regions of local energetic instability that may influence protein flexibility or interactions (a concept known as surface frustration), which could be conserved across family members (Derbyshire & Raffaele, 2023; Marín et al., 2013; Singleton & Eisen, 2024).

The repeated association of surface loop variation, SLiM or IDR acquisition, duplication, and tandemisation with SUSS effector families suggests they represent recurrent evolutionary outcomes associated with the preservation of structural stability and diversification of host‐target interactions (Franceschetti et al., 2017; Seong & Krasileva, 2023). If so, each of these mechanisms appears compatible with preservation of the core SUSS fold required for protein stability and cellular delivery, whilst permitting surface‐level modifications that can redirect binding towards new host targets. The molecular mechanisms enabling SUSS diversification therefore appear to operate within strict evolutionary constraints tied to conservation of the core fold of each SUSS effector family. Intriguingly, these same constraints may also create predictable vulnerabilities exploitable for resistance engineering.

## THE WIDESPREAD PREVALENCE AND EVOLUTIONARY DYNAMICS OF SUSS EFFECTOR FAMILIES

The SUSS phenomenon extends beyond individual families to represent a widespread evolutionary strategy across diverse pathogen systems, with large‐scale computational analyses revealing the true scope of this pattern among fungal, protist and oomycete phytopathogens (Mukhopadhyay et al., 2025; Seong & Krasileva, 2023).

Structural genomics surveys using AlphaFold2 have analysed over 26 000 secreted proteins from 14 fungal phytopathogens, revealing that the majority of orphan effectors cluster into distinct structural families. This comprehensive analysis by Seong and Krasileva (2023) identified 62 conserved structure‐related families encompassing most candidate effectors, with individual families containing between 10 and 400+ members per pathogen species. This systematic approach provided the first genome‐wide investigation of what had been observed in individual effector families through earlier structural studies.

The largest SUSS family comprises 453 RNase‐like effectors (RALPHs) (Seong & Krasileva, 2023), with 426 members found in *B. graminis* alone, representing approximately 43% of this pathogen's predicted effector repertoire. Other major families include 128 hydrophobin‐like effectors in *Puccinia graminis*, 30+ Tin2‐like effectors in *Ustilago maydis*, and numerous smaller families distributed across multiple pathogen species (Seong & Krasileva, 2023). The over‐representation of some of these SUSS effector families demonstrates that SUSS evolution represents a dominant pattern for some pathogens rather than isolated exceptions.

Similarly, a study by Mukhopadhyay et al. (2025) identified SUSS effectors across a diverse suite of gall‐forming microbes, including the plasmodiophorids *Plasmodiophora brassicae*, *Spongospora subterranea* and *Polymyxa betae*, the oomycete *Albugo candida* and the fungi *Taphrina deformans*, *Ustilago maydis* and *Synchytrium endobioticum*. This research further validates the prevalence of these strategies in fungi and oomycetes whilst establishing the paradigm within protist pathogens.

Several families identified in this study meet the SUSS criteria, most notably the Mig1 family in *U. maydis*, which preserves clear structural homology despite pairwise sequence identities falling as low as a staggering 0.6%. Furthermore, novel SUSS families were characterised, such as the AvrSen1‐like cluster in *S. endobioticum*, which maintain a conserved RAYH motif and the CCG family in *A. candida*. In these instances, the pathogens appear to utilise specific structural features, such as the RAYH alpha‐helix or conserved disulphide bridges, to anchor the core fold architecture and allow for rapid surface evolution (Mukhopadhyay et al., 2025).

Intriguingly, the identification of a nucleoside hydrolase‐like fold, structurally homologous to the bacterial effector HopQ1 and conserved across fungi, oomycetes and protists, further reinforces the widespread nature of the SUSS effector strategy and hints at the possibility of this virulence strategy not being limited to eukaryotic phytopathogens (Mukhopadhyay et al., 2025).

**Figure 2:**
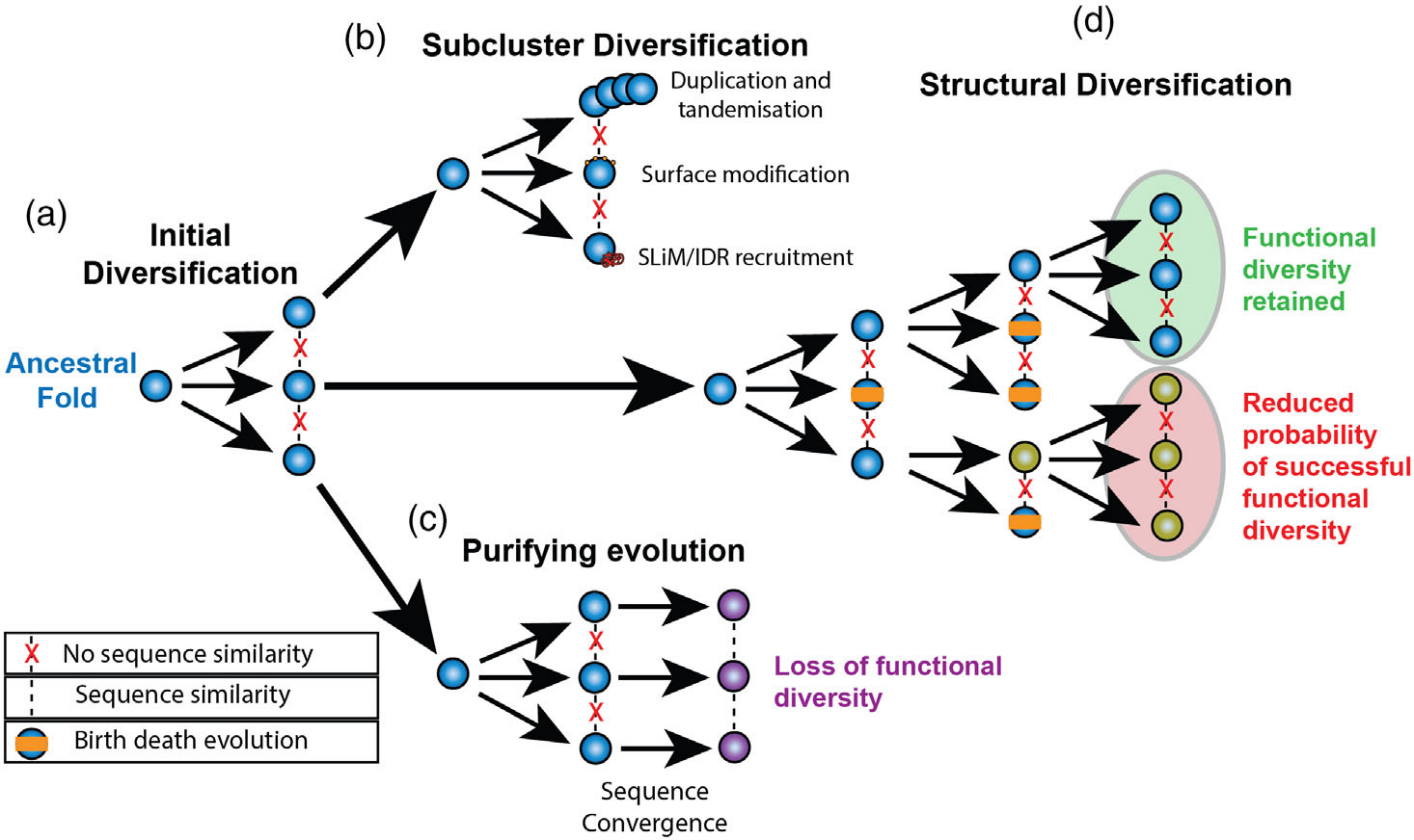
Potential evolutionary mechanisms behind SUSS effector diversification. Blue represents a retained core ancestral fold. Orange banners show birth–death evolution. (a) The core ancestral fold undergoes an initial diversification step where family members show loss of sequence similarity (Red cross). (b) Subcluster diversification represents a model SUSS expansion through the three common methods: surface modification, tandemisation and SLiM/IDR recruitment. (c) Clades might experience convergent evolution where a return of sequence similarity (Dashed lines) creates a fold that can no longer achieve the diverse functionality of the ancestral fold (Purple). (d) Structural evolution might diverge beyond the extent of the original core fold. If structural divergence becomes too extreme, these new novel structures will have unpredictable functionality and may lose the functional diversity shown in the original core fold (Yellow). Adapted from Seong and Krasileva (2023).

SUSS effector families within pathogen species exhibit rapid expansion driven by birth‐and‐death evolution (Figure 2). Following duplication, these gene copies are subjected to differential selection, resulting in either functional divergence, pseudogenisation or genomic deletion (Fouché et al., 2018; Latorre et al., 2020; Yoshida et al., 2016). *M. oryzae* isolates typically contain ~30 MAX effectors, organised into distinct subfamilies based on sequence similarity patterns (Seong & Krasileva, 2023). Similarly, *Phytophthora* species encode WY domain effectors as a part of their expanded RXLR repertoires, with evidence suggesting repeated duplication and divergence events that enable exploration of new host protein targets (Boutemy et al., 2011; Li, 2025).

Cross‐species comparisons reveal that SUSS effector families can span taxonomic boundaries whilst maintaining both structural conservation and functional targeting capabilities. The LARS fold identified in *L. maculans* also appears in *Fulvia fulva* effector Ecp11‐1, which triggers *Brassica* resistance despite the evolutionary distance between these pathogens (Derbyshire & Raffaele, 2023; Lazar et al., 2022). This is also demonstrated by the previously mentioned HopQ1‐like nucleoside hydrolase‐like fold from gall‐forming microbes, conserved across fungi, oomycetes and protists. Such wide taxonomic distribution underscores the evolutionary robustness of SUSS architectures, confirming their persistence as essential tools for diverse host targeting (Mukhopadhyay et al., 2025).

The existence of SUSS effector folds in unrelated taxa highlights that this phenomenon is likely universal among phytopathogens (Mukhopadhyay et al., 2025; Seong & Krasileva, 2023). It is not known how many SUSS folds exist, in part due to the high sequence diversity exhibited by effectors, making their identification through traditional genomic approaches challenging (Derbyshire & Raffaele, 2023; Seong & Krasileva, 2023). Recent structural genomics studies have laid a strong foundation to bridge this gap in our understanding, but the diversity of the structural landscape presents a significant challenge and will require on‐going research to understand the precise mechanisms behind the functional evolution of SUSS effectors in phytopathogens.

## SURFACE FRUSTRATION: A CONSERVED BIOPHYSICAL VULNERABILITY

The conservation of SUSS effector strategies across independent pathogen lineages suggests that once particular scaffolds become embedded in pathogen repertoires, variants that disrupt fold stability are selectively filtered, resulting in apparent evolutionary entrenchment of these scaffolds. An example of this may be the extreme diversification of RALPH/RNase effectors in *B. graminis*, which dominate the total effector secretomes of the pathogen as previously discussed (Seong & Krasileva, 2023). This entrenchment likely reflects the geometric precision required for fold stability, for example the tight packing of hydrophobic residues in the protein core, where small changes can propagate structural instability (Lim & Sauer, 1991), and this constraint may limit the configurational freedom of surface regions, despite their sequence variability.

**Figure 3:**
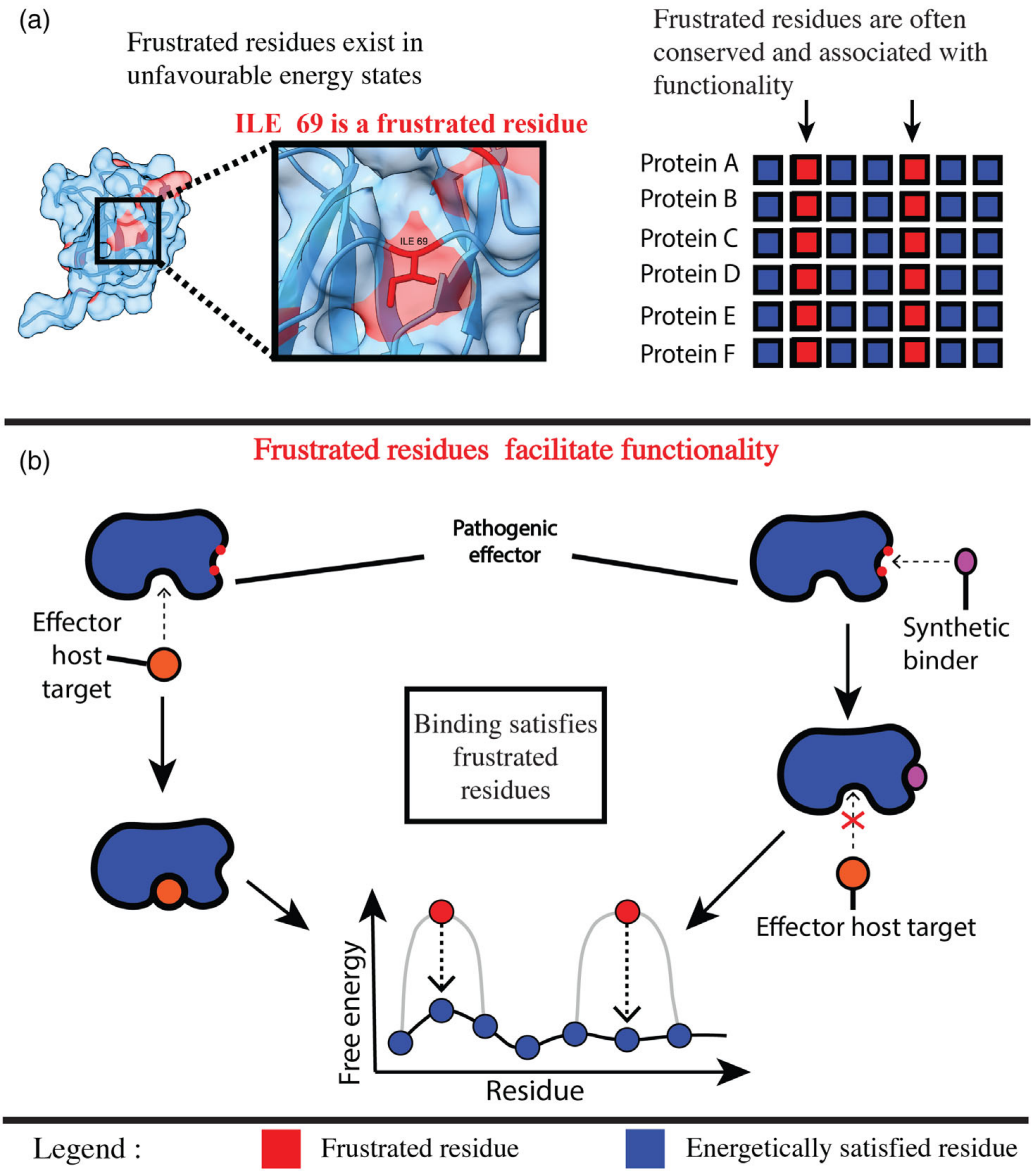
Protein frustration offers an opportunity to target effector function without explicit knowledge of the host target. Protein frustration offers the possibility to target effector functionality irrespective of host target. (a) Frustrated residues (shown in red) exist in higher energy states at their own expense to ensure the lowest possible energy conformation for the overall structure. New tools enable the computational identification of frustrated residues creating a highly efficient methodology in which effector functionality can be inhibited without any knowledge of the host target. The right‐hand side shows a representative sequence alignment of proteins of the same family. A blue box represents a residue that is not conserved. A red box represents a residue that is conserved. Frustrated residues are found to be conserved suggesting a role in functionality. (b) Pathogenic effectors contain frustrated residues that can be exploited to alter functionality. The left side shows the natural role that frustrated residues play in functionality, with their energy often lowered through important events such as binding of a host target. It is possible to leverage the role of frustrated residues by synthetically satisfying their energy levels using engineered binders. This could lower their energy levels, which would, in turn, prevent the critical functions such as host binding. The red arrows show how, in both cases, the frustrated residues achieve a lower free energy state.

To understand the nature of these constraints, it is necessary to introduce the biophysical concept of ‘frustration’. In protein folding, the majority of amino acids pack together to form stable, low‐energy interactions, following the ‘principle of minimal frustration’ to achieve a stable core (Ferreiro et al., 2018). However, specific regions of local frustration are recurrently observed and selectively retained, sites where residues are forced into energetically conflicting configurations, such as opposing electrostatic charges or steric clashes (Freiberger et al., 2021) (Figure 3a). These conserved regions of surface frustration are not folding errors, rather, they are frequently associated with functional sites. Much like a compressed spring within a rigid machine, these high‐energy pockets introduce localised flexibility into an otherwise stiff structure. This ‘energetic tension’ may facilitate the conformational flexibility required for binding interactions (Figure 3b) (Derbyshire & Raffaele, 2023; Ferreiro et al., 2018; Freiberger et al., 2023). The concept of surface frustration is best characterised in metamorphic protein families where high levels of surface frustration enable multiple stable conformational states (Freiberger et al., 2023), but has also been applied in PROTAC approaches for specific targeting of conserved protein features (Ma et al., 2025). Ma et al. demonstrated that frustration at protein–protein interfaces plays a central role in the cooperativity of PROTAC ternary complex formation, providing proof of concept that frustration patterns can be exploited for targeted molecular intervention.

In the context of SUSS effectors, this frustration is relevant for architectural tolerance. The SUSS folds appear to utilise frustrated patches as structural ‘shock absorbers’ that allow the stable core to support highly variable surface loops (Derbyshire & Raffaele, 2023). A concrete example is seen when comparing MAX effectors with divergent functions. AVR‐Pik and AVR‐Piz‐t share the core β‐sandwich fold but possess entirely different surface sequences, with AVR‐Pik binding HMA domain‐containing host proteins and AVR‐Piz‐t targeting a RING E3 ubiquitin ligase, respectively, yet computational analysis reveals that the underlying network of frustrated residues remains conserved in the same location relative to the core (Derbyshire & Raffaele, 2023). This indicates that the frustration is a property of the scaffold itself; a feature that may function as a hinge‐like element that tolerates the structural strain of diverse binding loops. Derbyshire and Raffaele (2023) performed computational analyses of 62 fungal effector families that revealed conserved frustration patterns despite extensive sequence divergence. Highly frustrated residues show significantly higher surface exposure versus minimally frustrated residues, and these regions correlate with predicted binding interfaces. Critically, frustrated regions cluster into distinct surface patches rather than distributing randomly, creating discrete vulnerable zones targetable by designed binding molecules. These patches likely represent convergent solutions to the biophysical requirements of engaging diverse host proteins, enabling intervention strategies that could disrupt multiple interactions simultaneously. The recurrent retention of surface frustration at these sites is consistent with a role in providing conformational flexibility essential for targeting multiple host protein families (Derbyshire & Raffaele, 2023).

The recurrent retention of surface frustration across SUSS effector families suggests it is associated with a fitness benefit, with observed conservation indicating that divergence from these patterns may incur fitness costs. Their flexible behaviour is thought to be associated with these locally unstable regions. This creates a targeting opportunity transcending sequence variation and specific host interactions. Surface frustration may be an Achilles' heel in SUSS effectors: eliminating frustrated residues risks losing the flexibility essential for diverse host protein targeting. Mutations reducing frustration decrease conformational plasticity, compromising the range of targets that defines SUSS effector success. Mutations increasing frustration beyond optimal levels destabilise the protein. This narrow evolutionary corridor has the potential to be exploitable through rational design.

## TARGETING OF SUSS EFFECTOR VULNERABILITIES WITH AI‐DRIVEN DESIGN STRATEGIES

Molecules, such as de novo designed protein binders, targeting frustrated surface patches may show activity against multiple SUSS family members whilst being less susceptible to effector sequence‐based escape. These molecules could function either by directly inhibiting effector activity or, if incorporated as sensory domains in immune receptors, by triggering broad‐spectrum immune recognition of the effector family (Figure 3b). Escaping such targeting would require fundamental changes to the SUSS effector scaffold itself and potentially compromise the ability of the effector to target diverse host proteins. Whilst it might be possible for single point mutations to escape recognition, we acknowledge that sequence variation at peripheral positions around frustrated residues could still disrupt interaction with individual designed binders—the key advantage is that escape would impose greater fitness trade‐offs than for conventional single‐target recognition. The identification of surface frustration as a conserved feature across SUSS effector families creates opportunities for rational design of broad‐spectrum resistance strategies that target the biophysical requirements for engaging diverse host protein families (Figure 3b). Unlike traditional approaches that focus on specific effector‐host protein interactions, frustration‐based targeting exploits fundamental structural constraints that are difficult for pathogens to escape without compromising their core virulence capabilities.

**Figure 4:**
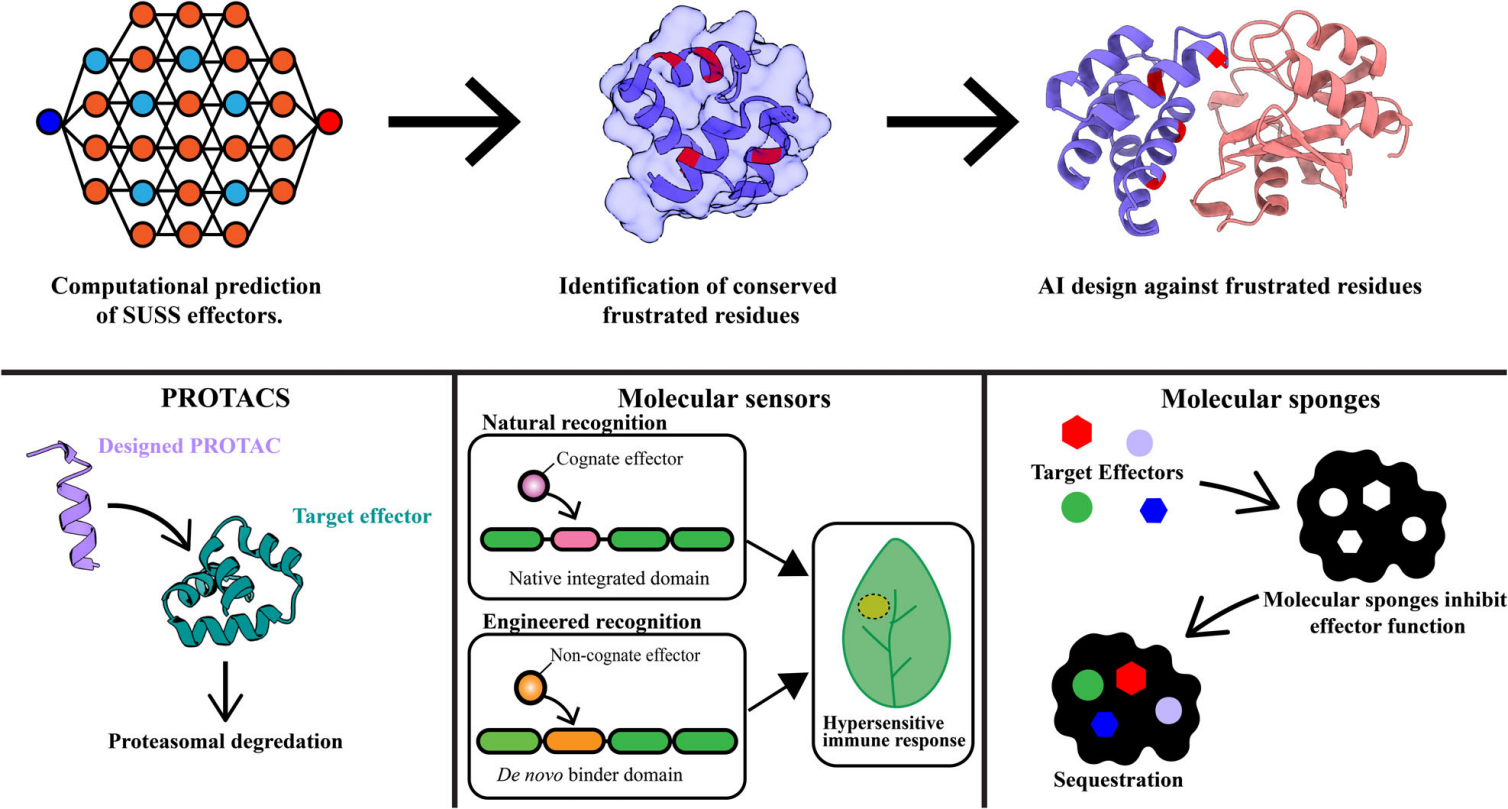
AI‐driven design strategies to target effectors for resistance bioengineering. (Top) A potential computational pipeline to engineer molecules using predicted frustrated residues. Initially the effector structures would need to be predicted using deep learning methods such as AlphaFold. This is represented by a neural network diagram. Following structural prediction, surface frustration would be characterised computationally. Frustrated residues are shown in red on the structure. Once identified, frustrated residues on the surface of effectors can be targeted using an AI protein design pipeline using programs such as RFdiffusion for backbone generation and ProteinMPNN for inverse folding to create *de novo* protein binders with specificity for the effector. (Bottom) Three potential applications of designed molecules. Designed PROTAC warheads recruit E3 ubiquitin ligase via fusion to target effector proteins for proteasomal degradation. Molecular sensors recognise non‐canonical effector proteins and trigger a cellular immune response. Molecular sponges inhibit effector activity by sequestration preventing activity on host targets.

New methods for AI‐driven protein design may now provide the computational tools necessary to translate frustration analyses into practical resistance strategies. Generative methods such as RFdiffusion can be utilised to design protein binders for specific protein surfaces (Kyro et al., 2025; Piochi & Khakzad, 2025; Watson et al., 2023). This may allow the creation of novel molecular interventions that bind to the frustrated regions of SUSS effectors associated with conformational flexibility, permitting the targeting of multiple host protein families (Figure 4). These designed binders would exploit the conserved geometric and energetic features of frustration hotspots rather than specific amino acid sequences or individual host protein binding sites, making them potentially robust to sequence evolution aimed at evading single‐target recognition (Derbyshire & Raffaele, 2023; Ma et al., 2025).

Three complementary targeting strategies emerge from this approach, each designed to disrupt the capacity for engaging diverse host proteins rather than blocking specific interactions (Figure 4). Molecular sponges represent soluble proteins designed to bind and sequester effectors before they reach any of their intended host protein targets (Distler & Tenzer, 2017; Langin et al., 2020). By targeting conserved frustration patterns, these sponges could potentially neutralise multiple SUSS family members with a single designed protein, regardless of which specific host proteins they individually target. Binders against pathogen effectors may also be incorporated as synthetic integrated domains into plant nucleotide‐binding leucine‐rich repeat (NLR) receptors (Zdrzałek et al., 2023). This may offer a more direct resistance mechanism than molecular sponges, potentially enabling broad‐spectrum recognition capabilities to the receptor, facilitating the detection of entire families. However, this approach risks auto‐immune activation through integration of novel domains that are incompatible with the immune receptor scaffold or improper activation through non‐specific/off‐target host interactions (Bentham et al., 2023). Finally, degradation warheads combine frustration‐binding domains with plant protein degradation machinery, functioning as PROTAC‐like molecules that redirect entire effector families for cellular turnover before they can engage their host protein targets (Su et al., 2024).

The host target agnostic nature of frustration‐based targeting provides potential advantages over traditional resistance engineering approaches. Current strategies require detailed structural and biochemical characterisation of specific effector‐host protein interactions—a time‐consuming process that yields narrow‐spectrum solutions effective against individual effector functions (Bentham et al., 2023; Maidment et al., 2023; Zdrzałek et al., 2024). Frustration‐based design can begin with the effector alone, enabling rapid development of countermeasures against effector families without requiring knowledge of their specific host protein targets or virulence mechanisms.

However, significant technical challenges remain. Frustration analysis depends on accurate structural models, yet disordered regions and surface loops that are most relevant for frustration and host protein interactions are often the least reliably modelled, creating the greatest uncertainty precisely where it matters most (Chen et al., 2020). Current generative design platforms, whilst promising, have limited benchmarking against plant pathogen effectors. This reflects not only the limited representation of plant pathogen effectors in the Protein Data Bank (PDB), but also the broader challenge of targeting conformational flexibility rather than static binding interfaces, a use case that remains systematically under‐evaluated in current pipelines. The use of AI models for the design and structural characterisation of proteins is still a nascent field and even the best models have significant caveats for their use (Koh et al., 2025). A key limitation is the accuracy of structural predictions for plant pathogen effectors used as protein design inputs. Current prediction models generalise less reliably to protein classes underrepresented in their training data, and plant pathogen proteins are particularly affected by this bias.

Despite these challenges, the potential advantages of AI‐based design for targeted disease resistance justify continued research. In particular, the broad targeting of SUSS effector folds would represent a significant evolutionary hurdle for the pathogen. The generative capacity of AI‐based pipelines may allow for the disruption / detection of multiple SUSS folds within a pathogen's repertoire, critically impairing pathogenicity and/or offering more durable protection than approaches that focus on the recognition of individual effectors.

## CONCLUSIONS

SUSS effector families represent a distinctive evolutionary outcome that enables plant pathogens to target diverse host protein families whilst maintaining the structural integrity required for effector delivery and activity. The systematic occurrence of these families across major pathogen groups demonstrates how conserved three‐dimensional scaffolds can serve as platforms for functional diversification towards different host cellular processes, challenging traditional assumptions about protein structure–function relationships.

The functional diversity of SUSS effectors begs the question: Do we put too much weight on structural homology for functional characterisation of effector families?

Current evidence demonstrates that SUSS effector family members maintain similar folds whilst engaging different host proteins, but our understanding of the mechanistic basis for this flexibility remains incomplete. Do frustrated surface regions enable promiscuous binding through conformational selection that allows engagement of structurally diverse host proteins, or do effectors employ distinct binding modes that happen to be compatible with the same structural framework? Understanding whether frustration patterns directly correlate with host protein binding versatility is crucial for predicting which surface features are truly conserved and targetable across diverse host interactions.

Finally, the integration of structural genomics, frustration analysis and AI‐driven protein design offers a conceptual framework for developing broad‐spectrum resistance that targets the fundamental capacity for diverse host protein interactions rather than individual effector functions. Unlike traditional approaches that focus on specific effector‐host protein pairs, frustration‐based strategies could potentially disrupt entire families' ability to engage their host protein targets through designed molecules that bind to the conserved structural features associated with conformational flexibility required for this versatility.

By targeting the recurrent structural constraints that enable diverse host protein targeting rather than individual virulence mechanisms, frustration‐based resistance could potentially provide protection against entire classes of effectors whilst imposing greater evolutionary trade‐offs on escape than current single‐target approaches, ultimately frustrating the pathogen's attempts at infection.

## CONFLICT OF INTEREST

The authors have no conflicts of interest to declare.

## Data Availability

Data sharing is not applicable to this article as no datasets were generated or analysed during the current study.
